# ANZCA at 25: past presidents in conversation

**DOI:** 10.1177/1329878X20941143

**Published:** 2020-11

**Authors:** Steven Maras

**Affiliations:** University of Western Australia, Australia

**Keywords:** ACA, ANZCA, Australia, communication, field, New Zealand

## Abstract

In 2019, the Australian and New Zealand Communication Association (ANZCA) celebrated its 25th anniversary. To commemorate this milestone, the organisers of the 2019 annual ANZCA conference in Canberra, Australia, convened a panel of past presidents involved in the transition of the Australian Communication Association (ACA), founded in 1980, into ANZCA. This article presents an edited transcript of that panel, with a pre-amble situating the panel in the context of current international research, with the dual purpose of marking an historical occasion, and also contributing to international research into the field.

In 1994, the Australian Communication Association (ACA), inaugurated in 1980, relaunched itself as the Australian and New Zealand Communication Association (ANZCA). In doing so, it embraced a vision of becoming an internationally relevant ‘trans-Tasman’ scholarly association.^1^ To commemorate the anniversary of this commitment to an expanded community and identity the organisers of the 2019 annual ANZCA conference in Canberra, Australia, convened a panel of past presidents directly involved in this transition. In the setting of the Museum of Australian Democracy, in Old Parliament House, the panel reviewed this transformational period, which coincided with a tumultuous time of change for communication and related discipline areas. This article comprises an edited transcript of that panel, informed by complementary research which aims to explore the significance of this kind of narrating of the field.

## Pre-amble

Written after the event it now attempts to frame, this pre-amble does not follow the typical convention of explicating the content of the panel, nor summarising the issues raised. Rather it seeks to shift the focus onto the scholarly and disciplinary ‘backdrop’ that forms an important, but invisible aspect of events that audiences typically encounter as ‘celebration’, ‘reflection’ or ‘conversation’ sessions in actual halls. While such events have a range of symbolic functions, and enact different ‘forms of capital’ to use Pierre Bourdieu’s (1986) term, my focus here on how a panel, such as ‘ANZCA at 25’, can assist making sense of the field as experienced (in a somewhat restricted sense) by the teachers and researchers working within it.

Recent attempts to think about the fields of media and communications study and research recognise that the area is at a key historical juncture. The disruption to ‘the discipline’ responds to a spiralling social significance for the subject matter; the structural transformation of higher education, and the rapid growth of interest in the field around the world (Simonson and Park, 2016: 2; also Fuchs and Qiu, 2018). Accounts, such as Peter Simonson and David W. Park’s authoritative *The International History of Communication Study*, adopt a spirited international and comparativist turn. Capitalising on intellectual currents of de-Westernisation, globalisation and cosmopolitanism – and against the tendency to see communication research as an exclusively American invention (Waisbord, 2019: 4) – recent scholarship de-centres the United States and re-frames the development of the field as a ‘transatlantic discourse’ that forms the basis for ‘multiple modernities across the world’ (Simonson and Park, 2016: 1). This zeitgeist corresponds to the broader vision that ANZCA itself represented in the 1990s in adopting an outward-facing perspective with globally relevant aspirations.

In this revisionist approach to the field, the past focus on ‘founding fathers’ (Schramm, 1997), generally white (Chakravartty et al., 2018), male, and based in the United States (Meyen, 2012), is eschewed in favour of a new project that recognises ‘a multiplicity of communication studies and lines of interaction, influence, and hegemony among them’ (Simonson and Park, 2016: 1). Recognition of an Antipodean contribution to the field remains, however, a work in progress. Given the self-admitted Anglophone bias of *The International History of Communication Study*, it is surprising to note that the Australian and New Zealand cases are barely mentioned, except in connection with the United Kingdom. This is not for want of a record of the media and communication project in this region, and its development. Suffice to say that a literature already exists that explores Australasian media and communication studies in general (see Cunningham, 2010; Flew, 2010; Henderson et al., 2010; Matheson, 2010; Molloy, 1990; Putnis et al., 2002; Rahkonen, 2007; Sinclair, 2010), and the history of ACA and ANZCA specifically (Maras, 2004; Ticehurst, 1989, 1992).

This article seeks to address such gaps in the international record by making visible the Australasian project, albeit in a partial and situated manner. Why it is important to do so can be explained through reference to three key areas of concern in current discussions about this disciplinary arena:

1. The identity of the field. As noted in ‘Ferment in the Field’ debates of the 1980s (*Journal of Communication* 33(3), 1983) – supported by issues, such as ‘The Disciplinary Status of Communication Research’ (34(3), 1993) and ‘Epistemological and Disciplinary Intersections’ (58(4), 2008) – and the revisiting of this debate 35 years later in the format of ‘Ferment 2018’; (Fuchs and Qiu, 2018), it remains difficult to posit a singular identity for the disciplines engaged in communications study and research. Crucially, Christian Fuchs and Jack Qiu (2018) observe that in the time between the 1983 and 2018 issues, ‘the question of critical research was much more peripheral, whereas the focus was more on the discipline itself and the status of its sub-fields, with less attention given to its larger social role’ (p. 225). In response to the flux surrounding questions of the field, Simonson and Park (2016) base their project around the history of communication study, and not the field per se (p. 2). Drawing on the term ‘communication studies’ in his work – which to some Australian ears may have a 1980s ring (see Fiske, 1982) – Silvio Waisbord (2019), who was editor of the *Journal of Communication* at the time of the ‘Ferment 2018’ issue, argues that the area is fundamentally ‘post-disciplinary’, which he takes to mean trans-disciplinary and complex in a manner that defies fixed boundaries. In this context, questions of identity undoubtedly become more complicated, and also arguably more urgent in terms of the need to publicly establish the purpose and ‘social role’ of the field.
2. A danger in focussing on the national at the expense of the trans-national, and what has been termed ‘methodological nationalism’ (Fuchs and Qiu, 2018: 221).^2^ Concerted steps are being made recent work to tackle trans-national modes of communication study systematically (Averbeck-Lietz, 2012: 4). Drawing on the work of Maria Löblich and Andreas Scheu (2011), Stefanie Averbeck-Lietz (2012) puts forward a model of research into paradigms that studies disciplines in terms of the interactions of biographies, ideas and institutions.^3^ The International Association for Media and Communication Research (IAMCR) has itself committed itself to documenting its international work through its History Commission (http://iamcr.org/node/7352; see also Prodnik, 2017).
3. The need to examine what Waisbord (2019) describes as the ‘institutional architecture of academic units, professional associations and journals’ (p. 8), which Waisbord sees as more important than a unitary ‘collective commitment to a common body of knowledge, questions, and debates’. Interestingly, despite reference to concepts of ‘milieu’ and ‘generations’, ‘educational institutions’ and ‘schools of thought’, journals and research communities, Averbeck-Lietz does not look specifically at the role of geographically located communication associations. This is not through an absence of studies at this level (see Meyen, 2012, 2014; Nordenstreng, 2008; Weaver, 1977; O’Donnell and van Heekeren, 2015).

These three areas provide a useful frame through which to read the ANZCA at 25 panel.

In relation to the identity of the field of media and communication studies, in the transcript that follows Bill Ticehurst is perhaps the strongest promoter of a foundationalist view of the discipline as providing a theoretical and epistemological base, or hub, for a broad range of dependent fields of thought. This is a perspective that Waisbord, for example, would want to challenge in terms of the fragmentation and hyper-specialisation of the field. Although the panellists, Warwick Blood, Shirley Leitch and Bill Ticehurst represent the broad areas that have had a major impact on ANZCA’s activities – journalism, public relations, organisational and management studies – there is also a wider story of how the ANZCA does ‘boundary-work’ around these areas (O’Mahony, 2013). ANZCA reconfigures itself on a regular basis, as constituent groups form different strands or communities of interest, while still retaining a link to a more general vision of communication study: a balancing act that ANZCA manages through diverse keynotes, multiple conference streams, and awards such as the Anne Dunn prize.^4^ ANZCA stands alongside, and shares, its academic spaces and constituent members with other associations – often engaged in their own projects of reinvention and refiguring – such as the Cultural Studies Association of Australasia (formed in 1990), the Journalism Education and Research Association of Australia (JERAA; formed in 1975), the Australian Teachers of Media (active since 1982) and the Public Relations Institute of Australia (formed in 1949), among other regionally based organisations and multiple international ones.

The connections between regional organisations and global ones is an area in which Ticehurst has intimate experience, as a key figure in securing the joint 1994 ANZCA–International Communication Association (ICA) conference. With the theme of ‘Communication and Diversity’, the 1994 conference ushered in the period which is the focus of this paper. As has been noted by Peter Putnis (1986), the Australasian invention of the communications field builds upon a variety of the US and European constructions of the field, wedging itself between multiple paradigms. As a consequence, ANZCA finds itself at the ‘confluence of European critical perspectives, which largely emerged in Russia and Germany, and had then spread to England, and the American managerialist, functionalist models’ (see Ticehurst below). Also significant in its ambition to be locally relevant and outwards facing, is the manner ANZCA was modelled on previous associations, such as ANZAM: the Australia and New Zealand Association of Management and ANZAAS: the Australia and New Zealand Association for the Advancement of Science. Indeed, ANZCA consciously draws on regional articulations of trans-nationalism, which themselves derive from older formations, such as the Australian and New Zealand Army Corps (ANZAC) tradition.^5^

While the transition of the ACA into ANZCA has a distinct link to the ICA (a link strengthened through the strong involvement of senior ANZCA figures, such as Terry Flew and Colleen Mills), the broader question of the interaction between Australasian organisations and global ones cannot be restricted to the ICA. A valuable piece of future research would capture the Australasian contribution to the IAMCR. Researchers, such as John Sinclair, Virginia Nightingale, Bill Bonney and Grant Noble, and others too numerous to mention, have been engaged with the IAMCR since the 1980s and indeed led sections and interest groups. The IAMCR conference was held in Sydney in 1996, with Virginia Nightingale as convenor. Since the 2000s Australian engagement with IAMCR has deepened with Naren Chitty, John Sinclair, Frank Morgan, Gerard Goggin, Elske van de Fliert and Pradip Thomas, all serving senior positions (John Sinclair, 10 June 2020, personal communication), which captures only partially a deepening and ongoing engagement, and only a few of the figures involved. It should be noted that Bonney, Noble and Goggin are former ACA/ANZCA presidents. ANZCA itself has recognised the significance of these links through the executive committee roles of IAMCR liaison, and ICA liaison.

As the coming together of ANZCA represented an outward looking focus at a point when communication technologies were creating new dispersed communities across the globe, ANZCA helped expand the sense of the field, and with it the aspirations of its members. In terms of the institutional architecture and dimensions of the field, the ANZCA at 25 panel demonstrates how the organisation has been – and still remains – integral to the careers of individual scholars and the developments of Australasian disciplinary fields more widely. Associations are important platforms for networking and collaboration, often across lengthy durations of time, with scholars learning from people they might never otherwise meet, and also gleaning general knowledge about key developments across different intellectual groupings and networks. How things get done in Associations can sometimes be remarkable in themselves. Peter Putnis recalls that

I did as president do some work on … ANZCA. And I remember getting a colleague of mine at Bond University, Jim Corkery, … to do a legal opinion on what needed to be done to the constitution. … I remember that ANZCA paid him, in, I think, three bottles of good-quality red wine, for that work. (Putnis, in Maras, 2003: 6–7)

As Ticehurst has recounted elsewhere on his work as the then-ACA President (1991–1992) to secure the ICA conference for Sydney for 1994:

The International Communication Association only goes overseas once every four years. We began to work on our profile with the ICA by organising panels at their conferences. To get the conference to Australia (and not Israel who were our competition) required a personal link with the ICA, so I had Bob Cox [Administrator] and Mary-Ann Fitzpatrick [then President] of the ICA out to Australia a couple of times. I had a video made about Australia and the development of the ACA. I was also approached by the *Electronic Journal of Communication*, through the ICA, to do a special issue on Australian communication studies [in 1993]. (Ticehurst, in Maras, 2005; see Ticehurst, 1993)^6^

He goes to suggest that

The time and place for all of this was right, for the ICA also needed to become more international and less North American. Around this time I was also involved in the International Federation of Communication Associations. (Ticehurst, in Maras, 2005)

In relation to New Zealand, Ticehurst felt it important to bring it into the association partly because of ‘economies of scale’, and also because of the contribution made by a number of New Zealand scholars already involved with the ACA, such as Frank Sligo and Margaret MacLaren: ‘I thought it was unreasonable for us to have the ACA, and not have New Zealand scholars recognised for their contribution in our structure and constitution’ (Ticehurst, in Maras, 2005). Ticehurst notes some resistance within New Zealand, specifically from the more vocationally focussed (in his view) New Zealand Communication Association.

Shirley Leitch, also one of the panellists below, recalled the emergence of ANZCA in a president’s session during the 2002 annual conference:

I became president for one reason and that was to assist in building the field of Communication in New Zealand. … When I first went to the University of Waikato in 1990, there were no Communication courses as such. There was one … course, which was called ‘Communication’, but was really just teaching … traditional Business/English …. So I set out, I guess, a little agenda. We started off with some courses. We moved from courses to a major in a Management degree. From that, we went to creating a whole department, and then eventually managed to get a Communication degree. At every step of the way there was a huge amount of resistance. Now, there is a kind of irony in all of this because I have been a major critic in New Zealand … of market-demand-led education. I see that as a very bad thing. But I have to say that Communication has been the beneficiary of that. When I put on the first Communication course at Waikato at third-year level I was told: ‘Well, you can have this course, but if you don’t get 20 people we’ll cancel it’. I got 70 in that first year, which made it, I think, the biggest optional third-year course in the degree. After that, there were no more comments of that nature. So, market demand actually created Communication at Waikato. (Leitch, in Maras, 2003: 11–12)

While following a pattern of achieving academic standing through market success, Leitch points to other factors in the development of ANZCA, specifically a sense of isolation:

I saw, though, that the worst thing that could happen to Communication was that we would become isolated. There are only eight universities in New Zealand, and they are good universities, but that’s about as many as in a medium-sized city in other parts of the world – very insular. There is a New Zealand Communication Association, and they do a good job, but I didn’t think that that was enough, and so I was a real supporter of the move to bring New Zealand into this association. (Leitch, in Maras, 2003: 12)

As well as addressing isolation, the broadening of the Association into New Zealand also widened its membership base and invigorated its conferences and journals. Since 1994, New Zealand has hosted the ANZCA conference six times (1998, 2005, 2008, 2011, 2015 and 2018), with the position of ANZCA President being held by a New Zealand-based academic in the year following the conference as per the constitution.

Leitch’s view of the situation in New Zealand forms a fruitful reminder of the importance of comparative research on the development of the field between Australia and New Zealand; a project that remains at a nascent stage (see Flew, 2010; Matheson, 2010). As Graeme Turner (1998) has noted, in Australia ‘distinctions between the disciplinary categories of Media Studies, Communication Studies and Cultural Studies have become quite blurred’. Does the same hold in New Zealand where media studies retains its own rhythms and journal, *MEDIANZ* (https://medianz.otago.ac.nz/medianz)? Similarly, the balance between UK and US influences in New Zealand communications scholarship seem differently struck, leading to stronger links with business and management schools and greater strengths in areas, such as organisational communication, as well as an early focus on applied, professional studies – with cultural studies not ‘carved out as a distinct academic discipline … as it has in, for example, Australia’ (Henderson et al., 2010: 28).^7^ Institutional pressures to publish internationally also figure here as a possible point of contrast (Henderson et al., 2010: 30).

The personal networks and experience of any researcher working in this area will shape as well as limit the object of study. In a previous discussion, I used the term ‘field-work’ to describe the way ANZCA both articulates the field, and reacts to developments within it: ‘Thinking about the history of ANZCA thus becomes linked to an idea of thinking about what kind of field-work ANZCA does’ (Maras, 2004: 34). This concept represents an attempt to move beyond an objectivist account of ANZCA in its field, to look at how the field itself works on the Association. In light of the revisionist approaches to the history of field discussed above, this approach needs to take into account new disruptions, flows and speeds of information. As Fuchs and Qiu (2018) suggest: ‘Communication studies is not happening in a vacuum. It responds to the vicissitudes in and outside academia. Pressed by swift transformations around the field, the pace of change has accelerated, often moving quickly in many directions’ (p. 220).

Just as architecture is made up of positive and negative spaces, the dynamic between the Association and the field thus goes more than one way. Panels such as the one recorded here provide a brief opportunity to reflect upon a period in time, and acknowledge the importance of different perceptions and perspectives on key disciplinary developments. In this case, all the panellists have had close experience with ANZCA’s predecessor, the ACA, and also firsthand experience of its transformation into ANZCA.

Warwick Blood is an Emeritus Professor at the University of Canberra. Warwick was President of ANZCA in 1994–1995. In 1994, he convened a joint conference between ANZCA and the International Communication in Sydney, Australia, building upon the work by Bill Ticehurst. He is a journalist by background, latterly looking at risk theory, communication and health issues. His work is nationally and internationally important, particularly around the reporting and portrayal of suicide and mental illness.

Shirley Leitch is a Professorial Fellow at the Australian National University, in the Australian Studies unit. Her disciplinary background is in public relations, and she has a special interest in science communication. Shirley was the first New Zealander to hold the ANZCA Presidency, in 1998–1999.

Bill Ticehurst was the President of the ACA in 1991–1992. He retired as an Associate Dean (Curriculum and Quality Enhancement) of the Faculty of Business Studies at the University of Technology, Sydney in April 2004, and was a key figure in internationalising the Association.

Lelia Green is a Professor of Communications at Edith Cowan University, and was the President of ANZCA in 2001–2002. Prior to her appointment at ECU, Lelia worked as a researcher, director and producer with BBC Television. She is best known for her research into children’s lives online, often working a part of international research networks.

## Panel

Lelia Green: I’ll start by asking each of the panellists to talk about a high point in their recollection of the 25 years of ANZCA.

Bill Ticehurst: Well, the high point has always been the good friends and the colleagues that I’ve made through ANZCA, and its predecessor, the ACA. I think the high point of my vice presidency and presidency was to travel to Miami, Florida to the International Communication Association [in 1991], and there make a bid for the ICA conference to be held in Sydney [which occurred in 1994]. That was a very interesting time. Subsequently, after that, I travelled to New Zealand, to the University of Waikato, and to the Auckland Institute of Technology, to talk about the idea about forming ANZCA at our 1991 conference, which interestingly bears some resemblance to the one we are having here.

Now, because there was no communication field in Australia really before 1970, it was dispersed across the other disciplines. Scholars, people would come from all over the place to form the communications studies disciplines in Australia. At my level, I’d been involved in ANZAM and ANZAAS both of which had communication discipline streams. Given that we had this new, vibrant, maturing communication discipline, I thought, ‘Why shouldn’t we have ANZCA?’ So, it was there, from my move, with the idea of forming ANZCA.

Shirley Leitch: Without doubt, being part of the formation of ANZCA was obviously the high point. I can’t overstate how important this was to the maturing of communication as a discipline in New Zealand. At that time, there were scholars who were studying in the field of communication, but they were scattered, there were hardly any degree courses, there were a few majors. At Waikato, there was only a small group of communication scholars when Bill first came to visit. I think ANZCA gave us a professional association – it gave credibility – and that was really important. Back in the early 1990s, you can’t overstate how isolated things still were in New Zealand. So that was a real high point. The other thing that I think I would like to recognise about ANZCA is how much it’s always embraced gender equality. At a time when most other academic organisations were very male-dominated, and the women were very much on the outer, women and men have always had equal footing within this organisation, and there have been many women presidents as well as male presidents. For women scholars coming through in an era when there was still an awful lot of prejudice, when it was still very difficult as a woman to make your way in academia, again, I can’t overstate the importance of the role that ANZCA played for me, personally, and for a lot of other women.

Warwick Blood: Well, let me just second the comments that have been made so far. I think it’s the friendship in ANZCA and its [predecessor the] ACA, that’s always been important to me: plus the mentoring, [and] the networks that are created among scholars. For me, though, the high point was definitely [the joint conference in Sydney]. Bill failed to mention that he and a colleague, Professor Bruce Molloy, were once seen at Los Angeles customs with two large cardboard boxes full of tiny koala bears, which were given out to all the people at the current ICA conference [then] to promote the idea of coming to Sydney, to have a joint conference. What a success it was. It was a great conference, particularly because of the joint sessions we had between ANZCA, newly formed, and ICA. That conference is something I’ll always remember, particularly because of the new friends that I created among some of our American scholars, and some of those networks have continued to this day.

Lelia Green: ICA … [in 2020] will be held in the Gold Coast, having been brought to Australia by Terry Flew, another ex-president of ANZCA. So the tradition continues. So, Bill, what would you say might be ANZCA’s past contributions over the past 25 years to the discipline, or to any other area of life?

Bill Ticehurst: I have to go back to the nature of disciplines and disciplinarity. There is nothing sacred about particular disciplines, whether it be English, journalism, metaphysics, the natural sciences, mathematics, calculus. They’re simply the organisation and structuring of knowledge into forms that are convenient to help us understand the world. In the 1970s, a new field emerged, a new discipline structured from old disciplines: so there was a fracturing and restructuring of old knowledge sets, which helped us better understand the world. This new ‘discipline’ was based around the notion of communication, which is necessary for us, as sentient human beings, to move from being objects, to being subjective, social beings. Without communication, we’re isolates. The communication discipline, then, provided us with a theoretical foundation of knowledge around which applied fields, such as journalism, public relations, or communication management, health communication and similar fields, could have a theoretical and epistemological basis for research and theory building.

I think the other thing that facilitated the emergence of the field in Australia and New Zealand, which was happening around the world, which Peter Putnis [1986] has written about, is that by accident of history, Australia and New Zealand were at the confluence of European critical perspectives, which largely emerged in Russia and Germany, and had then spread to England, and the American managerialist, functionalist models of communication. So, in ANZCA, we’ve married those disciplines, and epistemologically we are a very catholic organisation. I think those are the important things about ANZCA. [There’s] a need for us; we provide a central focus. Whenever I talk to anyone who is undertaking doctoral research, or a Master’s degree, or talk about their papers, the questions I always ask are, ‘Why is this meaningful? What are the theoretical foundations underlying the questions you’re asking?’ And ANZCA is centred around these questions, with its satellite … centres of enquiry.

Lelia Green: Thank you. And Shirley, I find it hard to believe because there was so much interesting work coming out of New Zealand in the 1990s that you thought it was really just beginning when you started with ANZCA, but tell us what you think that last 25 years of ANZCA engagement has done? I know it’s incredibly enriched the Australian scholarship when I think about people that have come [through the organisation] ….

Shirley Leitch: Well, I think building on what Bill has said, 25 years ago people didn’t have PhDs in communication. My PhD is in political science. If you actually looked at most of the people who first took up lectureships in communication, they had degrees in other fields, and now they have degrees in communication, and that has been quite fundamental. I can still remember celebrating at Waikato when Judy Motion got the first PhD in the area of public relations in the whole country. So that’s been a huge step. So it’s not just been a discipline that has been interdisciplinary and very much invited contributions from a number of other fields, it’s become a field in its own right, and a very rich and deep one that, again, as Bill has said, formed a whole lot of off-shoots as well.

There was a lot of depth at Waikato, and looking back on it I’ve always been kind of surprised by the maturity of a lot of the scholarship that was coming out of that little group, but I think that was kind of serendipity and one of those happy accidents of history. …

Warwick Blood: What illustrates what Bill and Shirley are talking about, I think, is the development of both graduate, postgraduate, courses in communication. That’s what ANZCA has supported, and many of its members have led. You can clearly see that in the two awards that ANZCA has: the Grant Noble Award honouring one of our earliest presidents, Grant Noble, and of course the Anne Dunn Award, honouring the late Anne Dunn. If you look at those awards – just go to the website those of you who are new members – and just look at the recipients of those awards. It’s like looking at a list of ARC grant holders. I mean, they’ve all become national or international researchers. As I look out here …, I can see some of them …. They’ve all done so very well in their field, and that’s what ANZCA has supported. We’re talking about the development of communication as a discipline.

Lelia Green: … What would you see, Warwick, as being the challenges and the opportunities facing ANZCA now as we look forward?

Warwick Blood: I think one of the challenges is to build up the profile nationally. I mean, a public profile. I know a lot of you are doing that through social media, through appearances just on traditional media. But I think that’s very important for us. I mean, I’d like to see more ANZCA members, presidents, vice-presidents, specialists in some areas, on radio, on television, putting the case forward. Because I think, often we get a lot of pretty inane, silly comment from a lot of people who think they know something about communication. …

One of the other challenges is there seem to be more and more conferences. Certainly, I get them on my email, and I think, ‘Who is organising this? Where are you from?’ So a conference like ours, which is quite broad in its scope and very inviting and inclusive, needs protecting. That’s why the theme of the conference becomes very, very important, and a good example of course is the theme for this conference [, ‘Making Sense: Data, Publics and Storytelling’], which is absolutely excellent given the times.

Lelia Green: So, Shirley, if you were looking at opportunities and challenges for ANZCA, things we could be doing better, things that we perhaps shouldn’t have done, or shouldn’t be doing?

Shirley Leitch: I think one of the big challenges for ANZCA is to continue to build the profile of the scholarship and particularly of the journals. There’s a newer journal now within ANZCA, and there’s a major job to really build the international profile of that. I think these days with rankings of journals so critical, and with academics being very much pushed into publishing only in journals with high impact factors, one of the things ANZCA can do to help all communication scholars is to help strengthen and build those journals.

Lelia Green: Bill? …

Bill Ticehurst: … What should ANZCA be doing now? More of the same, except there is such a fertile communication field in front of us. Our society and our community is replete with opportunities for research. … Fake news. Its consumers. Fake news is real news to those people. What is their world? What are their meanings? What are the implications of professional journalists no longer being the gatekeepers of knowledge, of information that’s given to directly to individuals? The consumers of this knowledge are no longer having it filtered through professional journalists. They’re getting raw knowledge, and by and large, many people can’t discriminate between what’s fiction and nonsense, and real news. So there’s all sorts of issues in terms of communication education, communication research. …

Communication has become more and more important than it ever was. It is the essence of our existence. And to be quite frank, it’s worrying the hell out of us, and having all sorts of implications; probably for the survival of humanity on earth if we don’t start getting it straight in science communication, credibility studies, all sorts of related issues. So ANZCA is an essential and critical organisation, with lots and lots of opportunities and hard work and discovery lies ahead.

Lelia Green: Shirley, given that, do you think that we should be more public facing? As communication specialists, should we be out there in the public saying, ‘We need to communicate this’. Should we be more publicly engaged in these controversial areas?

Shirley Leitch: ANZCA could become much more publicly engaged. There are so many opportunities for an organisation like ours to engage with some of the really big and important issues of the day. For example, you only have to think about the *Christchurch Call* in the context of the Christchurch massacre [in New Zealand], and the Online Harms Bill that was rushed through the Australian Parliament just before it was disbanded [for the 2019 election]. I think that these sorts of issues centre on communication, and there are huge communication dimensions to them. It’s not just a matter of laws and regulation, it’s also a matter of understanding what communication is and how people make sense of the world, and I believe ANZCA has a central role there.

Warwick Blood: That just extends what I was trying to say earlier, that the public profile is important, and I think there are issues where ANZCA can stand up and say, ‘Hey, this is what we can contribute to this issue, or to this debate’.

Lelia Green: So Bill, what would you be your wish for the future for ANZCA?

Bill Ticehurst: To rid the world of the notion that the sending of messages is necessary and sufficient for communication to take place, and that we need to start looking at communication as a meaning creation process, and conceptualising our satellite disciplines as such. Second, I want ANZCA to be for all its members, as fulfilling, as rich, and providing the opportunities for satisfying a thirst after knowledge, and the professional satisfaction, that I’ve had by being part of this organisation.

Lelia Green: Shirley, what would you see as a wish for the future?

Shirley Leitch: Well, I hope that ANZCA continues to be a force for good in the lives of people who are interested in the study of communication. I think it has been a sustaining force and place for development of several generations of scholars now. I look around the room and I see new faces, but also many faces who have been with ANZCA for quite a long time. And I think that the organisation is always going to be driven by the generation who occupy the executive chairs at that particular point in history, and those people are the ones who put their hands up. So, I’m just hoping that those who are currently putting their hands up are people who really understand just how important communication is now in the world. We used to think back in the 1990s that the real problem was the concentration of media in the hands of people like Rupert Murdoch. Now we understand that if you are looking for more diversity of media and of messages, be careful what you wish for. So again, it’s all about the driving force that is the current executive, the driving force that is the current membership of ANZCA, and just continuing to have those really important conversations as an organisation.

Lelia Green: Warwick?

Warwick Blood: Well, obviously we want ANZCA to thrive and be strong, as it has been over the years. I mean, one of the things that’s important in all of the nice words we’ve said, is to look back sometimes at what communication actually meant. Communication history is very important, particularly for our students, and often that gets neglected in some of the curricula around the place.

Lelia Green: That’s probably a place for me to hand over to our current president, Mary Simpson. So thank you very much.

Mary Simpson: I’d just like to thank each of our past presidents for reminding us that the past is as relevant today as it was then, 25 years ago, and I’m just going to tell a little story. Shirley Leitch signed off my enrolment when I returned as a mature student in my late 30s. I enrolled in a postgraduate diploma in communication management, no less, and I still remember being scared going into her office. So some things don’t change between students and teachers. So, I hope that all of you, those new members and the recent arrivals, and all of those who have been with us for a long time, take heed of the blast from the past, and enjoy it.

## Notes

1. The Tasman Sea, colloquially known as ‘the Ditch’, separates Australia and New Zealand.

2. Past claims for the desirability of an ‘Australian’ communication studies could indeed be seen in these terms, but also carried a high awareness of cultural cross currents (see Maras, 2004: 16).

3. Terry Flew (2010: 6) suggests that a historical rather than spatial approach to the shaping of distinctive national communication frameworks may lead us to a more careful consideration of issues such as the rise of cultural studies, the ‘cultural policy moment’, Internet and media convergence, debates around professionalism and secondary school media education.

4. Space does not allow for a close analysis of the contributions of ANZCA keynotes addresses to shaping the field. However, Stuart Cunningham (2010) notes presentation of some of his first work on cultural policy and innovation at the annual ANZCA conference in 2002 (p. 15) and Helen Wilson (2001) contribution formed a significant intervention into non-binary accounts of the field (Wilson, 2001).

5. This is not to efface differences between Australia and New Zealand as members of the Commonwealth of Nations with defining ties to the British Empire. Henderson et al. (2010: 32) have documented specific issues faced by *Pākehā* New Zealand researchers in addressing a *kaupapa Māori* approach, while Matheson (2010) has identified some of the gaps that have arisen in New Zealand due to the size of its funding bases and structures.

6. A description of how the ICA was held at Brisbane in 2020 would be different. While noted, this account would require attention that is not possible here.

7. One avenue of investigation could be the contribution of US scholars working in New Zealand. Organisational psychologist George Hines played a key role in introducing organisational communication at Massey University from 1979 (Frank Sligo, 17 May 2020, personal communication). Ted Zorn, formerly of Waikato University (1996–2012) and now at Massey, held the same Deanship as Hines did (2012–2018).
